# Update on the cardio-vascular adaptation at birth

**DOI:** 10.1186/1824-7288-41-S1-A32

**Published:** 2015-09-24

**Authors:** Graeme R Polglase, Stuart B Hooper

**Affiliations:** 1 The Ritchie Centre, Hudson Institute of Medical Research, Clayton Victoria 3168, Australia; 2 Department of Obstetrics and Gynaecology, Monash University, Clayton Victoria 3168, Australia

## Background

Worldwide, millions of babies are born each day, and in many of these infants, the umbilical cord is severed and they must begin air-breathing to survive. These events dramatically change the infant's circulation, transforming it from the fetal to the postnatal form, which then persists for the rest of its life[1,2]. However, if an infant is not breathing at birth, umbilical cord clamping will reduce venous return to the infant's heart (preload) by ~50% and increase systemic vascular resistance (afterload); both of which decrease cardiac output[3]. Cardiac output will remain low until breathing commences, when it triggers the increase in pulmonary blood flow needed to restore preload for the heart[4].

We have recently discoveredthat commencing ventilation before the umbilical cord is clamped, which is arguably the most natural sequence of events, stabilizes the circulation and avoids the loss and then restoration in cardiac output after birth[5]. Further, we demonstrated that it improves systemic and cerebral oxygenation by preventing the infant from becoming hypoxic during the transition at birth [6].

Our current research aims to examine how the infant's position, above or below the placenta, and uterine contractions induced by oxytocin administration, influences umbilical blood flow and the distribution of blood between infant and placenta during delayed umbilical cord clamping (DCC) at birth.

## Methods

All studies were approved by Monash University animal ethics committee. At 0.7days gestation, preterm lambs were delivered and instrumented for measurement of umbilical, cardiovascular and cerebral pressures and flows. Blood volumes were measured beforeand after DCC using biotin-labeled red blood cells. Lambs were placed 10cm above or 10cm below the midline of the ewe and ventilation commenced. The umbilical cord was clamped 3 minutes after ventilation onset and lambs ventilation continued. In a separate group, oxytocin was administered to the ewe (I.V. 20IU) during DCC.

## Results

Gravity had no effect on cardiopulmonary haemodynamics. Placing lambs below the placenta reduced UA and UV flow compared lambs placed above the placenta, resulting in increased pulmonary blood flow. No significant difference in blood volume was detected. There was no difference in systemic or cerebral oxygen kinetics during the transition at birth. Oxytocin administration during DCC has significant effects on umbilical blood flow and causes decreased arterial and cerebral oxygenation.

## Conclusion

Management of the mother and baby during DCC can influence oxygenation and the cardiovascular transition at preterm birth.
